# University of Pennsylvania

**Published:** 1848-09

**Authors:** 


### UNIVERSITY OF PENNSYLVANIA.

From the annual report of the Faculty, we learn that George W. Norris, M. D., has been appointed Professor of Clinical Surgery, vice Dr. Jacob Randolph, deceased. This is a good appointment. Dr. Norris is one of the Surgeons of the Pennsylvania Hospital, where his lectures will be given, and to which all who take the ticket of the Hospital will have access.

